# Molecular dissection of the valproic acid effects on glioma cells

**DOI:** 10.18632/oncotarget.11379

**Published:** 2016-08-18

**Authors:** Sabine Hoja, Markus Schulze, Michael Rehli, Martin Proescholdt, Christel Herold-Mende, Peter Hau, Markus J. Riemenschneider

**Affiliations:** ^1^ Department of Neuropathology, Regensburg University Hospital, Regensburg, Germany; ^2^ Department of Internal Medicine III, Regensburg University Hospital, Regensburg, Germany; ^3^ RCI Regensburg Centre for Interventional Immunology, Regensburg University Hospital, Regensburg, Germany; ^4^ Department of Neurosurgery, Regensburg University Hospital, Regensburg, Germany; ^5^ Wilhelm Sander Neuro-Oncology Unit, Regensburg University Hospital, Regensburg, Germany; ^6^ Experimental Neurosurgery, Department of Neurosurgery, University of Heidelberg, Heidelberg, Germany; ^7^ Department of Neurology, Regensburg University, Regensburg, Germany

**Keywords:** brain cancer, glioblastoma, temozolomide, HDAC inhibitor, SLC transporter

## Abstract

Many glioblastoma patients suffer from seizures why they are treated with antiepileptic agents. Valproic acid (VPA) is a histone deacetylase inhibitor that apart from its anticonvulsive effects in some retrospective studies has been suggested to lead to a superior outcome of glioblastoma patients. However, the exact molecular effects of VPA treatment on glioblastoma cells have not yet been deciphered. We treated glioblastoma cells with VPA, recorded the functional effects of this treatment and performed a global and unbiased next generation sequencing study on the chromatin (ChIP) and RNA level. 1) VPA treatment clearly sensitized glioma cells to temozolomide: A protruding VPA-induced molecular feature in this context was the transcriptional upregulation/reexpression of numerous solute carrier (SLC) transporters that was also reflected by euchromatinization on the histone level and a reexpression of SLC transporters in human biopsy samples after VPA treatment. DNA repair genes were adversely reduced. 2) VPA treatment, however, also reduced cell proliferation in temozolomide-naive cells: On the molecular level in this context we observed a transcriptional upregulation/reexpression and euchromatinization of several glioblastoma relevant tumor suppressor genes and a reduction of stemness markers, while transcriptional subtype classification (mesenchymal/proneural) remained unaltered. Taken together, these findings argue for both temozolomide-dependent and -independent effects of VPA. VPA might increase the uptake of temozolomide and simultaneously lead to a less malignant glioblastoma phenotype. From a mere molecular perspective these findings might indicate a surplus value of VPA in glioblastoma therapy and could therefore contribute an additional ratio for clinical decision making.

## INTRODUCTION

Glioblastoma multiforme is the most common malignant brain tumor. The standard therapy consists of surgical removal and concomitant radiochemotherapy followed by adjuvant chemotherapy with temozolomide [1]. But despite this aggressive procedure, the prognosis remains poor. Since 20-50% of glioblastoma patients suffer from seizures [2], antiepileptic drugs (AEDs) are often administered alongside of chemotherapy and might influence the effect of the chemotherapeutic agent or exert direct chemotherapy-independent effects on the tumor itself.

Valproic acid (VPA), for example, is an antiepileptic drug that is often used to treat seizures in glioblastoma patients. Interestingly, a number of retrospective analyses supported a disease course-modifying role of valproic acid in glioma patients [3–9]. As such also in the EORTC/NCIC temozolomide registration trial for glioblastoma, patients receiving VPA showed a prolonged survival over those receiving other (enzyme-inducing) or no AEDs.

Several cell biological studies have investigated VPA effects on tumor cells *in vitro.* It could be demonstrated that VPA inhibits cell proliferation by causing cell-cycle arrest in the G1 and/or G2 phase and that it induces differentiation and/or apoptosis in cancer cells [10, 11]. Valproic acid also reduced proliferation rates in glioblastoma-derived stem cells [12] and decreased cell viability of primary human glioblastoma cells [13]. Furthermore, valproic acid was suggested to downregulate the expression of *MGMT* (O-6-methylguanine-DNA methyltransferase) and to sensitize human glioma cells to temozolomide and irradiation [14, 15].

Nevertheless, there is yet little systematic understanding of the exact VPA mode of action and -in particular- the molecular correlates associated with it. We thus treated a large cohort of classical adherent glioblastoma and primary glioblastoma stem cell lines with VPA, recorded the functional implications of this treatment and performed global and unbiased next generation sequencing (NGS) analysis on the RNA level. As VPA functions as a histone-deacetylase inhibitor (HDACi) and specifically inhibits HDAC classes I and IIa [16] we also performed chromatin immunoprecipitation followed by NGS (ChIP-Seq) comparing VPA-naive with VPA-treated glioblastoma cells. We thus aimed to decipher how VPA alters the epigenetic decor of the tumor cells.

Despite a recent metaanalysis of prospective clinical trials that could not confirm a beneficial effect of VPA on outcome in newly diagnosed glioblastoma patients [17], there is currently an ongoing debate on whether the putative beneficial VPA effects in glioblastoma should be tested in a prospective randomized clinical trial [18]. Our study may inform on the drug's mode of action in glioblastoma cells and thereby provide an additional molecular ratio for clinical decision making.

## RESULTS

### Valproic acid sensitizes glioma cell lines to temozolomide and decreases proliferation

**Figure 1:**
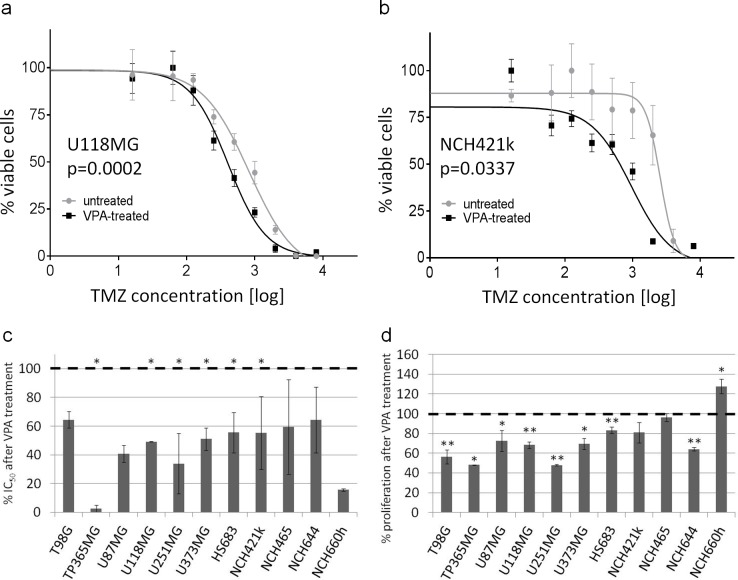
VPA causes sensitization to temozolomide and reduced proliferation (**a**, **b**, **c**) Valproic acid leads to a sensitization to temozolomide in seven adherent and four stem cell lines. Shown is the relative reduction of IC_50_ values (*p<0.05 in at least one experiment) after VPA treatment (c) which results in a shift of the temozolomide response curves to the left, exemplarily shown for U118MG (a) and NCH421k (b). (**d**) Valproic acid also leads to (significantly) reduced proliferation rates in most of the analyzed cell lines. *p<0.05, **p<0.01; two-tailed t-test. Results were reproduced in a second independent experiment.

First, we aimed to investigate the functional implications of VPA treatment on glioma cells. We therefore treated the six established glioblastoma cell lines and HS683 with VPA and subsequently performed temozolomide chemosensitivity assays. All seven cell lines were sensitized to temozolomide (Figure 1a, 1c). Five cell lines (TP365MG, U118MG, U251MG, U373MG and HS683) even showed a statistically significant VPA-induced reduction (p<0.05) of their IC_50_ values.

We then subjected the seven glioblastoma stem cell lines to identical experimental conditions. However, three of the lines, i.e. NCH1425, NCH601 and NCH636, were not suited for functional assays due to the fact that they were very sensitive to dissociation procedures necessary for cell counting and seeding. Hence, they were omitted from the experiment. In the four remaining stem cell lines that could be tested, we observed a similar sensitization to temozolomide after VPA treatment as for the established glioma cell lines (Figure 1b, 1c).

We next wanted to know whether VPA treatment would have effects on tumor cell proliferation also irrespectively of temozolomide in a chemotherapy-naive situation. Indeed, in the broad majority of the investigated cell lines (with exception of NCH421k, NCH465 and NCH660h) VPA treatment significantly reduced proliferation (two-tailed t-test: p<0.05 or <0.01, respectively; Figure 1d).

### Unbiased bioinformatical analysis suggests activation of multiple SLC transporters as a main molecular correlate of VPA response

**Figure 2:**
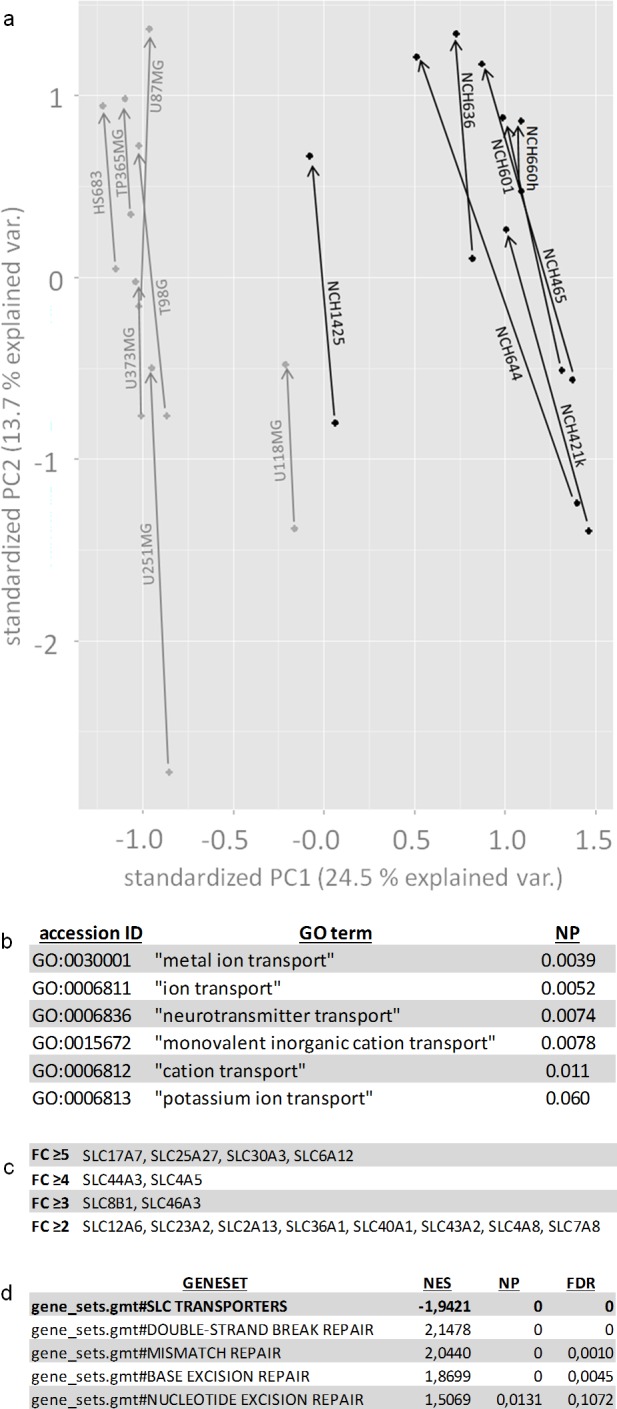
Bioinformatical analyses of VPA response signatures (**a**) Principal component analysis (PCA) reveals that VPA similarly affects gene expression in all investigated cell lines by a shift on the PC2 axis (indicated by the arrows). The PC1 axis segregates cancer stem cells from adherent glioblastoma cell lines. The stem cell line NCH1425 maps relatively close to the adherent cell lines which reflects the fact that -instead of typical sphere formation- this cell line exhibits a more adherent growth mode than the other stem cell lines. (**b**) GO term analysis with the 497 gene list (upregulated in ≥4 out of 7 adherent and ≥4 out of 7 glioblastoma stem cell lines, p<0.01, FC ≥5) demonstrates significant enrichment of several GO terms containing the term “transport” (p<0.1). (**c**) SLC transporters both with significantly enhanced H3ac-promoter-binding and significant transcriptional upregulation (≥4 out of the 7 adherent and ≥4 out of the 7 glioblastoma stem cell lines, p<0.01). Transporters are listed by fold change differences (FC ≥5, FC ≥4, FC ≥3, FC ≥2) according to RNA-Seq. (**d**) Gene set enrichment analysis confirms the upregulation of SLC transporters and additionally discloses a downregulation of different groups of DNA repair genes (double strand break repair, mismatch repair, base excision repair and nucleotide excision repair). For exact definition of gene sets compare Table S4. NP = normalized p-value, FC = fold change, NES = normalized enrichment score, FDR = false discovery rate.

In order to gain insight into the molecular correlates of the VPA effect on human glioma cells we analyzed all 14 cell lines before and after treatment with valproic acid by RNA-Seq and ChIP-Seq. Using RNA-Seq, we performed principal component analysis (PCA) based on the RPKM (reads per kilobase per million mapped reads) values of the sequenced samples (Figure 2a). Samples on the PC1 axis were separated by cell type (i.e. established adherent vs. stem cell lines). On the PC2 axis, samples were separated by whether they had received VPA treatment or not. Of note, VPA treatment shifted all cell lines in the same direction irrespective of whether they were adherent or stem cell lines arguing for concordant molecular VPA effects in both cell types. We then analyzed the transcriptome data from all cell lines by performing a pairwise comparison (VPA-treated versus untreated) for each individual cell line using the Audic-Claverie algorithm [19]. This led to the identification of a few thousand upregulated genes in each single cell line (adjusted p<0.01; Table S2). ChIP-Seq analysis revealed a few hundred thousand regions per cell line (enriched regions, p<0.01; Table S2) that had an increase of acetylated histone H3. These still corresponded to a few ten thousand euchromatinized regions with promoter correlation (−1000 to 0 relative to transcription start site). To further narrow down the number of target genes we analyzed all 14 cell lines and not only allowed for the detection of genes that were upregulated in all cell lines but also in the majority (at least 4 out of 7) of both the adherent and the stem cell lines. Applying the most conservative fold change cut-off (≥5), on RNA-Seq 497 genes met these criteria (Table S2).

This 497-gene expression signature was then subjected to GO term analyses. GO terms containing the designation “transport” were most prominently enriched (six different GO terms, adjusted p<0.1 each, Benjamini corrected, Figure 2b) and within these GO terms SLC transporters were the most prominent group of genes. SLC transporters were significantly enriched in the 497-gene list (16 transporters) as compared to the whole genome (46,111 genes, 397 SLC transporters; Fisher's exact test: p=1.18 E-5). 4 out of the 16 transporters were also epigenetically altered by promoter euchromatinization. The number of both epigenetically altered (euchromatinized) and overexpressed transporters increased with choosing less conservative fold change cut-offs (4 at ≥5, 6 at ≥4, 8 at ≥3, 16 at ≥2, Table S2, Figure 2c). Gene set enrichment analysis (GSEA) was then used to confirm the enrichment of the 16 SLC transporters that at fold change ≥2 were both overexpressed and epigenetically altered in our VPA-response signatures (normalized p<0.05) (Figure 2d).

### Validation of upregulation of SLC transporters in glioma cells and human patient biopsy samples after VPA treatment

We performed real-time RT-PCR analyses to validate the reexpression of SLC transporters in our glioma cell lines after VPA treatment. We exemplarily analyzed the four transporters (SLC17A7, SLC25A27, SLC30A3 and SLC6A12) that were upregulated on the mRNA level with the highest fold change (≥5) and had concomitant promoter euchromatinization on ChIP-Seq. All four transporters after VPA treatment were overexpressed in the majority of cell lines, partly up to more than 100-fold (Figure S1).

**Figure 3:**
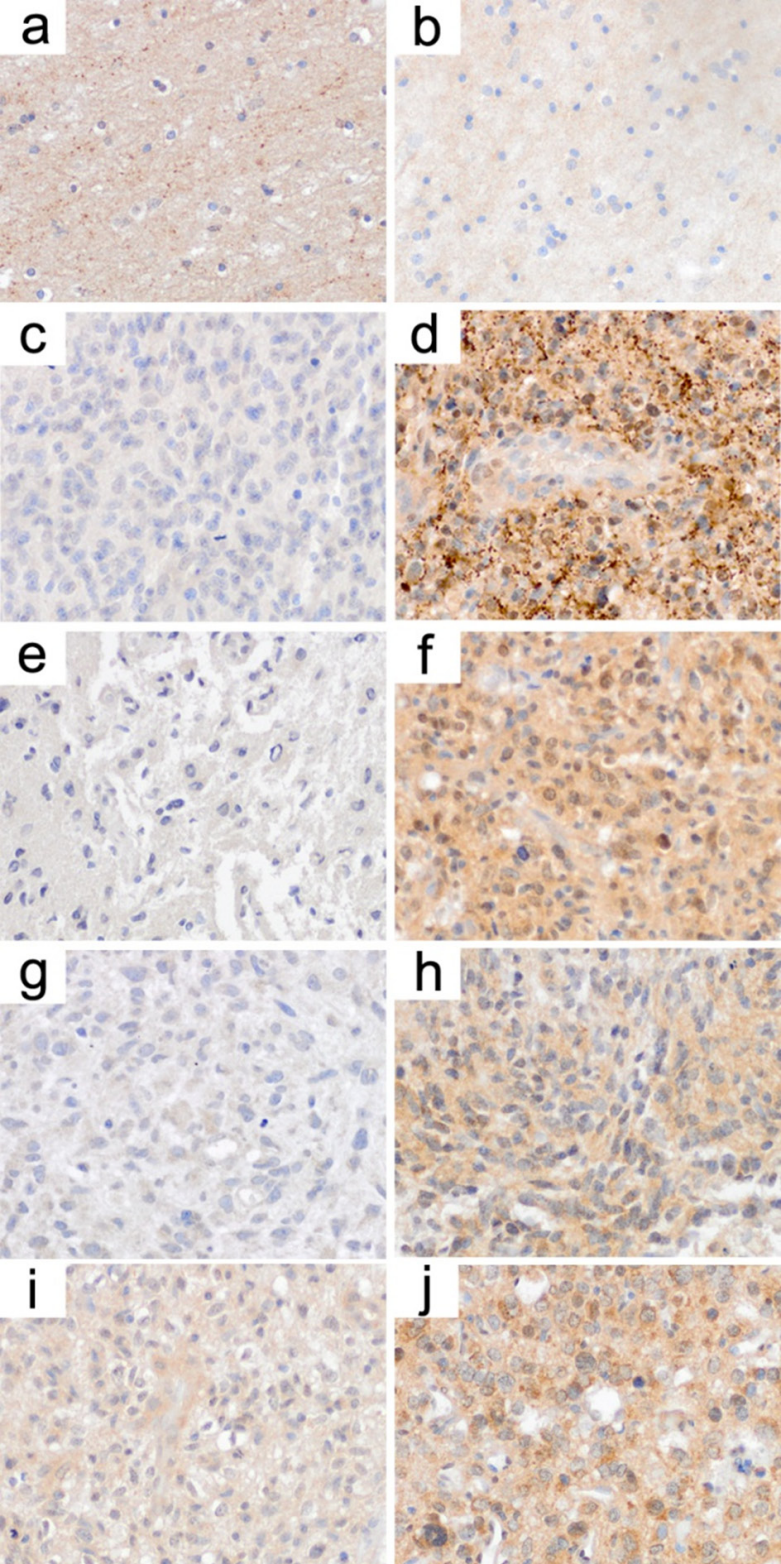
Immunohistochemistry states reactivation of SLC transporter expression in patients’ biopsy samples under VPA treatment SLC transporters are weakly expressed in non-neoplastic brain tissue with SLC17A7 (**a**) a little stronger than SLC25A27 (**b**). c/d, e/f, g/h, i/j; matched pairs of biopsy samples from individual patients before (**c, e, g, i**) and under (**d, f, h, j**) valproic acid treatment. c/d shows immunohistochemistry for SLC17A7, while e/f, g/h and i/j show immunohistochemistry for SLC25A27. Note that there is a clear increase in SLC transporter expression in the biopsy samples following valproic acid treatment. These effects were more consistently (higher fraction of patients) observed for SLC25A27.

For two of the transporters (SLC17A7 and SCL25A27) antibodies were available that worked in formalin-fixed paraffin-embedded tissue. We stained matched pairs of patient biopsy samples (n=6) that allowed for the comparison of SLC transporter expression prior and after VPA treatment. Indeed, for SLC17A7 we detected a reexpression in three out of six patients (one clear and two moderate increases, Table S3, Figure 3c, 3d). For SLC25A27, four out of six patients had an obvious reexpression. While in five patients the second biopsy was taken directly under VPA treatment, patient 4 was biopsied for the second time three years after VPA had been discontinued. Thus, in fact four out of five patients showed an increase in SLC25A27 expression after the drug's application (Table S3, Figure 3e-3j). Interestingly in this context, in non-neoplastic brain tissue SLC17A7 was expressed stronger than SLC25A27 (Figure 3a, 3b). These higher basal expression levels of SLC17A17 might explain for the fact that VPA-induced upregulation for this transporter was not as strongly and clearly visible as for SLC25A27.

### Functional implications of altered SLC transporter expression on glioma cells

We reasoned that if the overexpression of SLC transporters should account for the sensitization of VPA-treated cells to temozolomide, siRNA knockdown of SLC transporters should rescue the VPA-induced chemosensitization. We therefore performed siRNA knockdown experiments with the 16 transporters (Figure 2c) that were upregulated and euchromatinized following VPA treatment.

**Figure 4:**
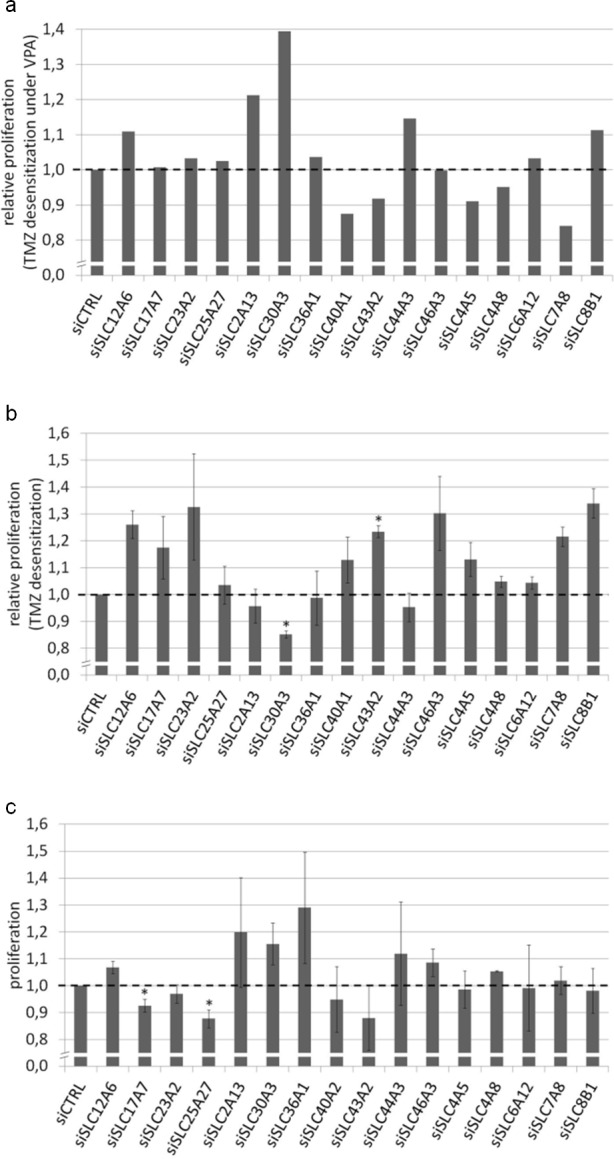
Functional effects of siRNA-mediated SLC transporter knockdown U373MG cells were transfected with siRNAs against 16 different SLC transporters and analyzed for proliferation using the Resazurin assay. (**a**) VPA and temozolomide treatment: The effect of siRNA-mediated SLC transporter knockdown on the temozolomide response was compared between VPA-treated and VPA-naive cells (relative proliferation difference between VPA-treated and VPA-naive siCTRL cells set to 1). For the knockdown of single transporters (such as SLC30A3) there was a weak functional reversal of the VPA effect corresponding to a relative TMZ desensitization under VPA. (**b**) Temozolomide treatment only: When assessing the temozolomide response independently of VPA, the knockdown for a higher number of transporters led to a slightly enhanced relative proliferation corresponding to temozolomide desensitization (relative proliferation between temozolomide-treated and temozolomide-naive siCTRL cells set to 1). (**c**) Neither VPA nor temozolomide treatment: There were no major direct effects of SLC transporter knockdown on tumor cell proliferation itself. *p<0.05, two-tailed t-test.

VPA-pretreated and -naive U373MG cells were transfected with the respective siRNAs or negative control siRNA. Then, temozolomide was applied in concentrations (1,500 μM) close to the expected IC_50_ doses. We first calculated the extent of VPA-induced sensitization in response to temozolomide (relative reduction of proliferation by temozolomide in the VPA-treated cells compared to the VPA-naive cells) for the siCTRL transfection and set this to 1. We then assessed the VPA-induced effects in response to temozolomide with the respective targeting siRNAs and normalized them to siCTRL. If for a defined siRNA the VPA-induced temozolomide sensitization was stronger compared to siCTRL this would lead to a relative proliferation value of <1 indicating an additional sensitization induced by the siRNA knockdown. If the VPA-induced temozolomide sensitization in the context of a defined siRNA was weaker compared to siRNA control this would lead to a relative proliferation value >1 indicating a desensitization by the respective siRNA knockdown and thus the presumed rescue effect (Figure 4a). Here, for the knockdown of single transporters (such as SLC30A3) we observed a weak functional reversal of the VPA effect. Nevertheless, the observed effects did not appear strong and consistent enough to fulfill the criteria of a successful rescue experiment.

Since this rather complex experimental setting did not allow for detecting a clear rescue effect we next wanted to know whether the SLC transporter knockdown in a situation uninfluenced by VPA would lead to temozolomide desensitization. This would be indicative for the transporters being involved in the uptake of temozolomide. Here, results were still rather inconsistent but knockdown for a higher number of transporters led to a slightly enhanced relative proliferation (temozolomide-treated versus temozolomide-naive, siCTRL set to 1; Figure 4b). This, particularly in consideration of the fact that SLC transporter knockdown did not have major direct effects on tumor cell proliferation itself (Figure 4c), might provide subtle indications for a relevance of individual SLC transporters in the VPA-mediated temozolomide response. In the discussion section we will further comment on the experimental limitations that may have impeded a higher clearness of the results.

### Other molecular factors that might explain for a better temozolomide response under VPA treatment

As MGMT had been linked to the VPA response [14], we assessed VPA-induced changes of *MGMT* expression, promoter methylation and promoter euchromatinization in our cell lines (Table 1a). Except for a significant upregulation (p<0.01) in one single cell line (NCH660h), mRNA expression after VPA treatment was largely unaffected. For *MGMT* promoter methylation only TP365MG showed a moderate increase (74 to 94%) after VPA treatment. H3ac binding to the *MGMT* promoter was significantly increased (p<0.01) in six cell lines but this cannot explain for temozolomide sensitization. Taken together, these findings do not argue for a major relevance of MGMT in the VPA response.

**Table 1:**
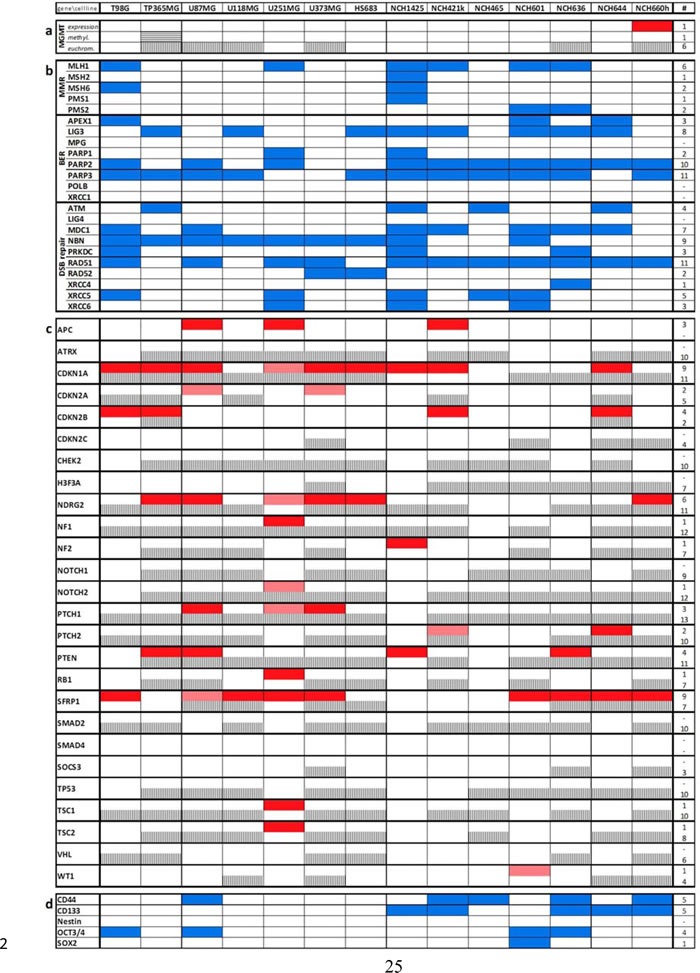
Synopsis of the different types of genes affected in their transcription or H3ac-promoter DNA binding levels by VPA treatment

MGMT (a) was investigated for overexpression, increase in methylation and euchromatinization; DNA repair genes (b) and stem cell markers (d) were investigated for transcriptional downregulation; and tumor suppressor genes were investigated for euchromatinization and transcriptional upregulation. Blue indicates that a gene is significantly downregulated and dark red that a gene is significantly upregulated on the mRNA level (fold change ≤2, p<0.01). Light red additionally highlights genes that are upregulated (fold change ≤2) but do not reach significance. Horizontal stripes indicate an increase in promoter methylation whereas vertical stripes indicate significant (p<0.01) promoter euchromatinization induced by VPA. Note that MGMT is rather activated than inactivated by VPA. Other DNA damage repair genes and stem cell markers are commonly reduced (b and d), while a number of established tumor suppressor genes gets euchromatinized and reexpressed by VPA (c).

Apart from upregulation of SLC transporters, GSEA also pointed towards a downregulation of DNA repair genes after VPA treatment (Figure 2d; Table S4). Euchromatinization of inhibitors of DNA repair genes could be a possible explanation for this phenomenon. We therefore analyzed in further detail the most important genes of the three functional groups “mismatch repair” (MMR), “base excision repair” (BER) and “double-strand break repair” (DSB repair) [20] (Table 1b). Within the group of base excision repair genes, *PARP2* and *PARP3* were most frequently downregulated by VPA treatment since they showed a significant downregulation (p<0.01) in ten and eleven out of the 14 cell lines, respectively. Amongst the double-strand break repair genes, *NBN* and *RAD51* exhibited a significant downregulation in the majority of cell lines (nine and eleven lines, respectively). The expression of mismatch repair genes in comparison (except for *MLH1*) was downregulated in much lower frequencies in only individual cell lines.

### Molecular correlates for potential beneficial temozolomide-independent VPA effects on glioma cells

As indicated above (Figure 1d) we also observed a direct chemotherapy-independent negative effect of VPA on the proliferation of glioma cells. Thus, aside from molecular alterations that might explain for a chemosensitization of glioma cells we analyzed our signatures for molecular alterations that might explain for a therapy-independent “benignization” of the tumor genotype. We first analyzed a set of the most common tumor suppressor genes for transcriptional upregulation and promoter euchromatinization after VPA treatment (Table 1c). Indeed, almost all cell lines, with the exception of NCH465, exhibited reexpression of at least one tumor suppressor arguing for a relevance of tumor suppressor upregulation in the VPA effect. Two genes, namely *CDKN1A* (*p21*) and *SFRP1* appeared to be majorly affected, showing significant upregulation (p<0.01) in eight cell lines, respectively. Reexpression of *CDKN1A* was validated by q-RT-PCR analyses (Figure S2). Promoter euchromatinization was also observed for the majority of the genes and was even more frequent than transcriptional reexpression.

We next wanted to know whether VPA treatment would reduce the expression of classical stem cell markers such as CD44, CD133, Nestin, OCT3/4 and SOX2 (Table 1d). Indeed, we found a significant downregulation (p<0.01, Audic-Claverie algorithm) of stem cell markers (in particular CD44 and CD133) in a number of cell lines. Unsurprisingly, in general, the reduction was more frequently observed in the stem cell lines arguing for a differentiation-inducing effect of VPA.

**Figure 5:**
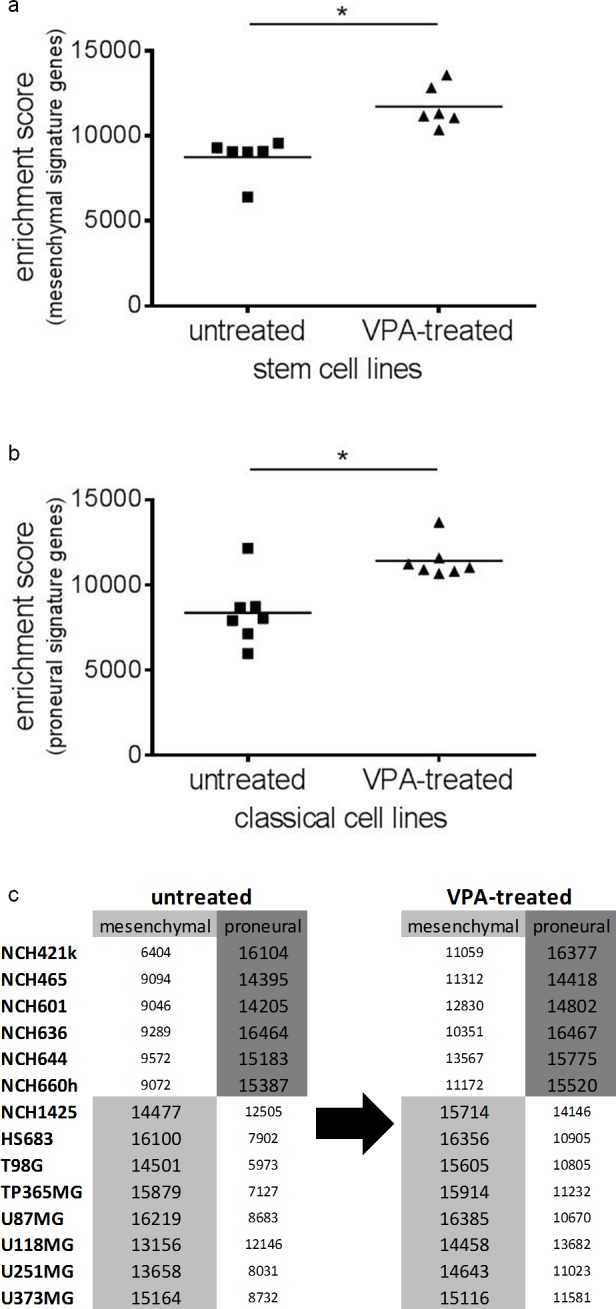
Gene-expression based molecular classification of the cell lines remains unchanged after VPA treatment Following VPA treatment, we observed a statistically significant enrichment (p=0.03) of mesenchymal signature genes in the glioblastoma stem cell lines (**a**), while in the adherent glioblastoma cell lines there was a statistically significant enrichment (p=0.02) of proneural signature genes (**b**). Nevertheless, VPA treatment did not change molecular subtype classification with the stem cell lines retaining the proneural and the adherent cell lines retaining the mesenchymal expression subtype as the prevailing one (**c**). *p<0.05, Wilcoxon signed rank test.

Finally, we investigated whether VPA treatment would change gene-expression based molecular classification of glioma cell lines (either mesenchymal or proneural) [21]. According to this classification all of our adherent glioma cell lines belong to the mesenchymal and all of our glioma stem cell lines (except for NCH1425) to the proneural expression subgroup. After VPA treatment the stem cell lines showed a significant enrichment of signature genes related to the mesenchymal subtype (Figure 5a; Wilcoxon signed rank test: p=0.03) while the adherent cell lines had a significant enrichment of proneural signature genes (Figure 5b; Wilcoxon signed rank test: p=0.02). This, however, did not change subtype classification of the respective cell lines. In the adherent cell lines the mesenchymal expression signature and in the stem cell lines the proneural expression signature still prevailed (Figure 5c). Thus, VPA treatment causes a general enrichment (particularly of weakly expressed) subtype-related signature genes but in its overall effects does not change the gene-expression based molecular classification.

## DISCUSSION

VPA, an antiepileptic drug used to treat seizures in brain-tumor patients, has been suggested to have anti-cancer effects but its exact molecular modes of action have not yet been deciphered. VPA is also a histone-deacetylase inhibitor [16]. We therefore investigated the molecular correlates of the VPA effect in glioma cells by using a comprehensive and unbiased genome-wide approach combining RNA-Seq and ChIP-Seq analyses.

First, we assessed the functional implications of VPA treatment on our cell lines. Indeed, in all lines studied VPA treatment sensitized glioma cells to temozolomide. This finding for adherent glioma cell lines had been reported in a similar fashion in a number of preceding *in vitro* studies [14, 15], but not for glioma stem-like cells.

Comparing the molecular profiles before and after VPA treatment we found that GO terms with the functional annotation “transport” were associated with the VPA response signature. Indeed, numerous SLC transporters were transcriptionally upregulated and euchromatinized following VPA treatment. The solute carrier (SLC) group of membrane transporters comprises a family of more than 300 membrane-bound transporters [22]. SLC transporters have also been shown to mediate the influx of cytotoxic drugs into cells [23] thereby increasing the intracellular drug concentrations. Also, they have been implicated in transporting anticancer drugs across the blood-brain-barrier [24]. Strikingly, when investigating matched patient biopsy tissue samples before and under VPA treatment we observed an increase of SLC transporters (SLC17A7 and SLC25A27) in most of the patients analyzed.

Given this apparent association between VPA-induced chemosensitization and SLC transporter upregulation we followed up functionally on these relationships. We first aimed to rescue the VPA-induced chemosensitization by siRNA-mediated transporter knockdown. Though leading to a moderate chemodesensitization for individual transporters these effects did not appear convincingly strong and consistent (Figure 4a). However, in a situation uninfluenced by VPA SLC transporter knockdown in a higher number of cell lines led to a slight chemodesensitization (Figure 4b) and SLC transporter knockdown did not appear to exert major direct effects on tumor cell proliferation itself (Figure 4c). Thus, overall these experiments might provide subtle indications for a role of SLC transporters in the temozolomide response. The potential causes that obscure more explicit effects are manifold: Firstly, as there are more than 300 different SLC transporters with overlapping function, the knockdown of a single transporter might simply not be sufficient to induce the desired effects. Secondly, though the siRNAs themselves had reasonable knockdown efficiencies (Figure S3a), the high SLC transporter expression increase induced by VPA might not have been restored to VPA-naive levels by siRNA treatment (Figure S3b). Functional responses would hence also be limited after siRNA treatment. Finally, temozolomide is known as a substance that due to its lipophilic properties and its relatively small size [25] can easily cross the blood-brain-barrier and also for intracellular uptake might not alone depend on uptake transporters like the SLCs.

As others had shown that VPA downregulated the expression of *MGMT* [14] and this could serve as an explanation for VPA-induced chemosensitization, we also explored this possibility. *MGMT* expression and promoter methylation were hardly altered by VPA treatment and the *MGMT* euchromatinization we observed could not explain for temozolomide sensitization. Taken together, our analyses do not support a major relevance of MGMT as mediator of the observed VPA response. In glioblastoma patients, however, also DNA repair mechanisms other than MGMT may modify the temozolomide response [20]. Of note, we detected a downregulation of BER and DSB repair genes as a common mechanism in a larger number of cell lines. Disruption of the whole BER mechanism and, in particular, downregulation of *APEX1* has been reported to enhance the cytotoxic effects of temozolomide [26, 27]. Also, PARP inhibitors have long been discussed for the treatment of glioblastoma patients [28, 29]. Inactivation of the DSB repair gene *NBN* resulted in a sensitization to temozolomide in melanoma cells [30] and the downregulation of *RAD51* induced a temozolomide sensitizing effect in glioma cells [31]. We found all these genes downregulated after VPA treatment (Table 1b). Downregulation of MMR genes, such as *MSH2* and *MSH6*, has been described to evolve under therapy with alkylating agents and would rather mediate opposite effects, i.e. temozolomide resistance [32, 33]. These genes (except for *MLH1*), however, were only reduced in individual cell lines.

Our functional analyses revealed that VPA treatment had also direct (temozolomide-independent) effects on tumor cell proliferation. A molecular correlate that might explain for this finding was the euchromatinization and upregulation of various well-established tumor suppressor genes, with *CDKN1A (p21)* and *SFRP1* being the most frequently upregulated genes on the mRNA level. The finding of *CDKN1A* reexpression is in line with a preceding publication that describes upregulation of this gene after VPA treatment [34]. *SFRP1* encodes a WNT inhibitor the expression of which has been shown to be of positive prognostic relevance in gliomas [35]. Both genes are also known to be epigenetically regulated in gliomas. VPA in this way might ameliorate the biological characteristics of glioma cells, inducing a benignization of glioma genotypes. VPA, particularly in the stem cell lines, also diminished the expression of classical stem cell markers (CD44, CD133) thus inducing a potential differentiation of glioma genotypes which is in line with a previous publication [12].

In summary, we here present a comprehensive molecular next generation sequencing study deciphering the global molecular VPA effects on the epigenetic (histone) and transcriptional level. We find that the VPA effects are manifold including molecular alterations that might directly enhance the temozolomide response and others that might induce temozolomide-independent favorable effects on glioma biology. Thus, our study from a molecular point of view highlights potential anti-tumorigenic modes of action of VPA in glioma cells. The question remains whether this also translates into relevant clinical effects as a very recent pooled analysis of prospective clinical trials (retrospective in nature in respect to VPA though) could not retrace an association between VPA use and improved survival outcomes in patients with newly diagnosed glioblastoma [17]. Factors that might impede a 1:1 translation of molecular into clinical effects are the multitude of molecular changes observed. We pointed out that on the level of each single cell line a few ten thousand euchromatinized regions with promoter correlation and a few thousand upregulated genes were observed (Table S2). As it is impossible to assess these alterations in their entirety, we concentrated on genes overlappingly regulated between cell lines. However, we cannot exclude that in individual cell lines/patients molecular changes might be effective that dilute the overall favorable response signatures explicated in our study (for example see our comment on the downregulation of MMR genes above). Also, in the framework of our work we were not able to cover additional pharmacokinetic effects that might modify VPA effects. In this regard, it has been reported, e.g., that VPA leads to a decreased oral clearance of temozolomide of about 5% [36].

In case a prospective randomized controlled clinical trial on the use of VPA in glioblastomas should still be intended, our analyses reveal that there a) will be no simple molecular read-out that could be used for stratification or an upfront identification of patients most likely to benefit from standard therapy plus VPA and that b) also the underlying molecular set-up of the individual patient may well influence the response towards the agent. For the study design it thus would be the challenge to best possibly control for such underlying molecular factors and to keep patient groups as homogeneous as possible.

## MATERIALS AND METHODS

### Cell lines and valproic acid (VPA) treatment

Six established adherent glioblastoma cell lines (T98G, TP365MG, U87MG, U118MG, U251MG and U373MG) and HS683 (derived from an anaplastic oligodendroglioma) were cultured under standard conditions (37°C, 5% CO_2_, DMEM, 10% FCS, 1% penicillin/streptomycin). In addition, seven human primary stem cell-like glioblastoma cell lines (NCH1425, NCH421k, NCH465, NCH601, NCH636, NCH644, NCH660h) [37], all of which were kindly provided by Professor Christel Herold-Mende (Dept. of Neurosurgery, Heidelberg University Hospital), were cultured under the following conditions: 37°C, 5% CO_2_, DMEM/Ham's F-12 medium, 1% penicillin/streptomycin, 1% L-glutamine. Directly before use, stem cell medium was prepared freshly by adding 20% BIT admixture supplement (Pelo Biotech, Planegg, Germany), 0.02% epidermal growth factor (EGF) (ReliaTech, Wolfenbuettel, Germany) and 0.02% basic fibroblast growth factor 2 (bFGF2) (ReliaTech). Valproic acid sodium salt (Sigma Aldrich, St. Louis, MO, USA) was dissolved in distilled water and subsequently diluted freshly in complete DMEM. Cells were treated with 7.5 mM VPA [38] for 36 h [39]. Origin of the cells was confirmed by short tandem repeat profiling prior to use either compared to known profiles (adherent cell lines) or to the original patient tissue (stem cell lines).

### RNA extraction and real-time reverse transcription (RT) PCR analysis

The RNeasy Plus Mini Kit (Qiagen, Hilden, Germany) was used according to the manufacturer's protocol to extract RNA from untreated and VPA-treated cells, respectively. Subsequent cDNA synthesis from one microgram total RNA was performed using random hexamer primers (Gene Link, Hawthorne, NY, USA) and the SuperScript^TM^ II Reverse Transcriptase (Life Technologies, Carlsbad, CA, USA). Expression of candidate gene transcripts was validated by real-time quantitative RT-PCR based on the SensiFAST^TM^ SYBR Hi-Rox Kit (Bioline, London, UK) with the StepOnePlus^TM^ sequence detection system (Life Technologies). Fold expression changes relative to non-neoplastic brain tissue were calculated with the ΔΔC_T_ method [40] using *GAPDH* (glyceraldehyde 3-phosphate dehydrogenase) as the reference transcript (primer sequences: Table S1).

### Chromatin immunoprecipitation (ChIP)

For chromatin immunoprecipitation 10^6^ cells (untreated and VPA-treated, respectively) were incubated with 1% formaldehyde (Roth, Karlsruhe, Germany) for 10 minutes to cross-link the DNA with proteins. Afterwards, cells were resuspended in freshly prepared swelling buffer. Isolated nuclei were then further processed using a commercial ChIP assay kit (Merck Millipore, Darmstadt, Germany) following the manufacturer's recommendations. After resuspension in SDS lysis buffer, lysates were sonicated with an ultra-sonicator (Covaris, Woburn, MA, USA) to shear the DNA into 200-800 bp fragments. Sonication was followed by centrifugation and samples were diluted in ChIP dilution buffer and precleared with salmon sperm DNA/protein A agarose beads. Immunoprecipitation with antibodies against acetylated histone H3 (H3ac) was performed overnight at 4°C, rabbit anti-human IgG fraction served as a negative control. Then, the antibody-histone-DNA complexes were collected and histone-DNA complexes were eluted in freshly prepared elution buffer. After reversion of the cross-link, the DNA was recovered by phenol/chloroform extraction and ethanol precipitation.

### Next-generation sequencing (NGS)

H3ac-immunoprecipitated DNA from untreated and treated cells was used to generate libraries for NGS using the TruSeq® ChIP Sample Preparation Kit (Illumina, San Diego, CA, USA) according to the manufacturer's instructions. Isolated RNA from untreated and VPA-treated cells was converted into libraries of template molecules suitable for NGS using the TruSeq® RNA Sample Preparation Kit v2 (Illumina). All libraries were quantified using the KAPA SYBR FAST ABI Prism Library Quantification Kit (Kapa Biosystems, Woburn, MA, USA). Equimolar amounts of each library were used for cluster generation on the cBot with the TruSeq SR Cluster Kit v3 (Illumina). The sequencing run was performed on a HiSeq 1000 instrument (Illumina) using the indexed, 50 cycles single read (SR) protocol and the TruSeq SBS v3 Kit (Illumina). Image analysis and base calling resulted in .bcl files which were then converted into .fastq files by the CASAVA1.8.2 software. Analysis of NGS data was performed using the Genomatix software (Genomatix, Munich, Germany). First, the .fastq files were mapped to the human genome hg19 (annotation based on ElDorado 12-2012) using the Genomatix Mining Station. Then, all unique hits were further processed using the Genomatix Genome Analyzer (Expression Analysis tool for RNA-Seq; ChIP-Seq Workflow and GenomeInspector tool [41] to identify significantly enriched promoter regions; List comparison tool to identify overlapping hits from RNA-Seq and ChIP-Seq analyses).

### Bioinformatical analyses using online bioinformatics resources

Principal component analysis (PCA) was based on the top 1,800 most variable genes that were calculated from the RPKM (reads per kilobase per million mapped reads) expression values. A biplot was then created with custom R scripts and the package ggbiplot. GO term analysis was performed using the DAVID (database for annotation, visualization and integrated discovery) bioinformatics resources as described elsewhere [42] and a corrected enrichment p-value for each GO term was calculated. Gene set enrichment analysis (GSEA; URL: http://www.broadinstitute.org/cancer/software/genepattern) was performed in order to determine whether a priori defined sets of genes were over- or underrepresented in our gene expression signatures. Glioblastoma signature genes were taken from [21].

### Temozolomide chemosensitivity assay and quantification of *MGMT* promoter methylation

Cells were seeded in triplicates in 96-well plates at a density of 500 cells in 100 μl per well. 24 h later, the medium was replaced by fresh medium with or without 7.5 mM valproic acid, respectively. After another 24 h, various concentrations of temozolomide (1–8,000 μM) were added. Cell viability was measured 72 h later using resazurin (R&D Systems, Minneapolis, MN, USA) as described elsewhere [43–45]. The half maximal inhibitory concentration (IC_50_) value was determined as the concentration resulting in a 50% growth reduction compared to control cell growth (i.e. cells that did not obtain temozolomide). Statistical analyses were performed using the GraphPad Prism software (version 6). Fluorescence intensities were log-transformed, normalized and then fitted with the methods of least squares and variable slope.

Methylation of *MGMT* promoter DNA from untreated and VPA-treated samples was assessed via MethyQESD (methylation-quantification of endonuclease-resistant DNA) as described before [46].

### siRNA transfections

Transfections with small interfering RNAs were performed using the DharmaFECT^TM^ transfection reagent (GE Healthcare Dharmacon, Freiburg, Germany) according to the manufacturer's protocol. Cells were seeded in triplicates in 96-well plates at a density of 3,000 cells in 100 μl per well. 24 h later, half of the cells were treated with 7.5 mM VPA for 24 h. Then, 25 nM of siRNAs were transfected in medium with or without valproic acid, respectively. The next day, temozolomide at a concentration of 1,500 μM was added and cell viability was measured 72 h later via the resazurin assay.

### Immunohistochemistry

Matched pairs of formalin-fixed and paraffin-embedded tissue samples from brain tumor patients before and after VPA treatment were selected from the tumor tissue archive of the Regensburg Department of Neuropathology and investigated according to protocols approved by the institutional review board. Immunohistochemical staining was performed following a standard protocol [35]. Briefly, 4-μm sections were cut, slides were deparaffinized and, after sodium citrate buffer antigen retrieval and blocking, incubated with the primary antibody for 45 minutes. As primary antibodies, we used anti-SLC17A7 and anti-SLC25A27 (both from Origene, Rockville, MD, USA). The EnVision^TM^+ Dual Link System-HRP (Dako by Agilent Technologies, ­Santa Clara, CA, USA) was used for detection of antibody binding according to the manufacturer's protocol. Afterwards, nuclei in the immunostained sections were counterstained with haematoxylin.

## SUPPLEMENTARY MATERIALS FIGURES AND TABLES


