# Supplementary material for: Molecular dissection of the valproic acid effects on glioma cells
Source: Oncotarget. 2016 Aug 18;7(39):62989–3002. doi: 10.18632/oncotarget.11379 (PMC5325342; doi:10.18632/oncotarget.11379)
Supplement: Supplementary file 1 [file oncotarget-07-62989-s001.pdf]

Molecular dissection of the valproic acid effects on glioma cells

Supplementary Material

Table S1: **Primer sequences used for expression analysis performing real-time RT-PCR**.

gene name	forward primer	reverse primer
**CDKN1A/p21**	5’-GCAGACCAGCATGACAGATTT-3’	5’-AAGATGTAGAGCGGGCCTTT-3’
**GAPDH**	5’-CATGACAACTTTGGTATCGTG-3’	5’-GTCCACCACTGACACGTTG-3’
**SLC2A13**	5’-GTCAGGTCAGAACGCCACTT-3’	5’-CTTGTAGCAGAAACCGCAGTC-3’
**SLC6A12**	5’-CTTGCCTGGGCTCTCTTCTA-3’	5’-TTCAGAAAGTCCGTGCAATG-3’
**SLC17A7**	5’-TGGGGCTACATTGTCACTCA-3’	5’-CTGAGGGGATCAGCATGTTT-3’
**SLC25A27**	5’-TGAATACACCACTTGAGGACAA-3’	5’-TTTGATGACATCGGCTGGT-3’
**SLC30A3**	5’-CTGCAGAGCTTTGGGGTACT-3’	5’-AAGGGCACAGATGGAGAAGA-3’
**SLC44A3**	5’-CGGCATGGTTATTCCTGTTC-3’	5’-ACATGTTGCCAAAGCTGTCA-3’

Table S2: **Overview on the number of H3ac-enriched promoter regions, transcriptionally upregulated genes and an overlap of both after VPA treatment.** Provided is the number of regions/genes per cell line using different fold change cut-offs (≥2, ≥3, ≥4 and ≥5). P-value cut-off is <0.01 for all comparisons. Additionally, at the bottom of the table the number of genes and SLC transporters is displayed that are upregulated in at least four out of the seven adherent and four out of the seven glioblastoma stem cell lines.

cell line	ChIP-Seq		RNA-Seq				list comparison ChIP & RNA (FC ≥ 2)	list comparison ChIP & RNA (FC ≥ 3)	list comparison ChIP & RNA (FC ≥ 4)	list comparison ChIP & RNA (FC ≥ 5)
	enriched regions (p<0.01)	Promoter correlation -1000 to 0 bp	upregulated genes (p<0.01, FC ≥ 2)	upregulated genes (p<0.01, FC ≥ 3)	upregulated genes (p<0.01, FC ≥ 4)	upregulated genes (p<0.01, FC ≥ 5)				
**T98G**	78,539	7,578	3,234	2,183	1,753	1,508	802	499	380	310
**TP365MG**	586,695	39,909	3,284	2,202	1,795	1,572	2,038	1,324	1,067	929
**U87MG**	759,187	53,630	3,780	2,631	2,127	1,813	2,570	1,723	1,375	1,178
**U118MG**	533,322	33,245	4,149	2,855	2,281	1,952	2,356	1,588	1,269	1,085
**U251MG**	130,235	9,973	5,532	3,752	2,871	2,362	1,953	1,289	954	769
**U373MG**	1,008,547	60,082	3,095	2,159	1,769	1,524	2,240	1,536	1,253	1,086
**HS683**	630,964	42,730	2,719	1,800	1,461	1,271	1,811	1,161	920	793
**NCH1425**	103,423	5,064	3,658	2,461	1,965	1,677	530	352	262	212
**NCH421k**	577,244	25,497	4,196	3,000	2,516	2,234	1,833	1,246	1,030	915
**NCH465**	201,424	14,905	3,375	1,990	1,449	1,176	1,294	731	517	413
**NCH601**	555,889	35,143	4,147	2,889	2,325	2,032	2,501	1,724	1,359	1,181
**NCH636**	441,038	31,005	2,653	1,686	1,288	1,076	1,512	921	687	566
**NCH644**	453,295	27,450	4,863	3,356	2,771	2,441	2,647	1,762	1,421	1,245
**NCH660h**	725,521	48,981	3,278	2,111	1,678	1,434	2,217	1,395	1,105	940
upregulated in ≥ 4 of 7 adherent and in ≥ 4 of 7 stem cell lines			1,607	863	623	497	379	178	114	87
upregulated SLC transporters			48	23	19	16	16	8	6	4

Table S3: **Immunhistochemical staining scores for the six matched pairs of patient biopsy samples before and under VPA treatment**. Ranges for staining intensities arise from heterogeneous tissue samples. Numbers correspond to 0=no/minimal, 1=weak, 2=moderate/intermediate and 3=strong staining intensity. †, biopsy in recurrence not taken under but three years after VPA treatment. VPA doses provided are daily doses.

patient no.	sex	before VPA				VPA dosis	under VPA		
		age	diagnosis	SLC17A7	SLC25A27		diagnosis	SLC17A7	SLC25A27
1	f	26	anaplastic oligoastrocytoma (WHO-III)	3	1	1,500 mg	anaplastic oligoastrocytoma (WHO-III)	3	3
2	f	67	glioblastoma (WHO-IV)	1	0	300 - 1,200 mg	glioblastoma (WHO-IV)	1-2	3
3	f	27	astrocytoma (WHO-II)	2	1	2,000 mg	anaplastic oligoastrocytoma (WHO-III)	1	2
4	f	39	anaplastic astrocytoma (WHO-III)	2	2	300 - 1,300 mg	anaplastic astrocytoma (WHO-III)†	0-3	0-2
5	m	65	glioblastoma (WHO-IV)	0-1	0-2	900 - 1,300 mg	glioblastoma (WHO-IV)	3	3
6	m	31	oligoastrocytoma (WHO-II)	3	3	600 mg	anaplastic oligoastrocytoma (WHO-III)	2	2
CTRL	m	64	non-neoplastic brain tissue	1-2	1				

Table S4: **Lists of genes used for gene set enrichment analysis (GSEA)**.

SLC TRANSPORTERS: SLC17A7, SLC25A27, SLC30A3, SLC6A12, SLC44A3, SLC4A5, SLC24A6, SLC46A3, SLC12A6, SLC23A2, SLC2A13, SLC36A1, SLC40A1, SLC43A2, SLC4A8, SLC7A8.
DOUBLE-STRAND BREAK REPAIR: BLM, BRCA1, BRCA2, CHEK1, DMC1, FEN1, H2AFX, HUS1, LIG1, LIG4, MDC1, MLH1, MRE11A, NBN, PRKDC, RAD21, RAD50, RAD51, RAD51B, RAD51C, RAD51D, RAD52, RAD54L, RPA1, TP53BP1, XRCC2, XRCC2, XRCC3, XRCC4, XRCC5, XRCC6.
MISMATCH REPAIR: ABL1, EXO1, MLH1, MLH3, MSH2, MSH3, MSH4, MSH5, MSH6, PCNA, PMS1, PMS2, POLD3, TP73, TREX1.
BASE EXCISION REPAIR: ADPRT1, APEX1, APEX2, CCNO, FEN1, LIG1, LIG3, MBD4, MPG, MUTYH, NEIL1, NEIL2, NEIL3, NTHL1, OGG1, PARP1, PARP2, PARP3, PCNA, PNKP, POLB, RPA1, SIRT1, SMUG1, TDG, UNG, XRCC1.
NUCLEOTIDE EXCISION REPAIR: ATXN3, BRIP1, CCNH, CDK7, DDB1, DDB2, ERCC1, ERCC2, ERCC3, ERCC4, ERCC5, ERCC6, ERCC8, LIG1, MMS19, NTHL1, OGG1, PCNA, PNKP, POLL, RAD23A, RAD23B, RPA1, RPA3, SIRT1, SLK, TP53, XAB2, XPA, XPC, XPD.

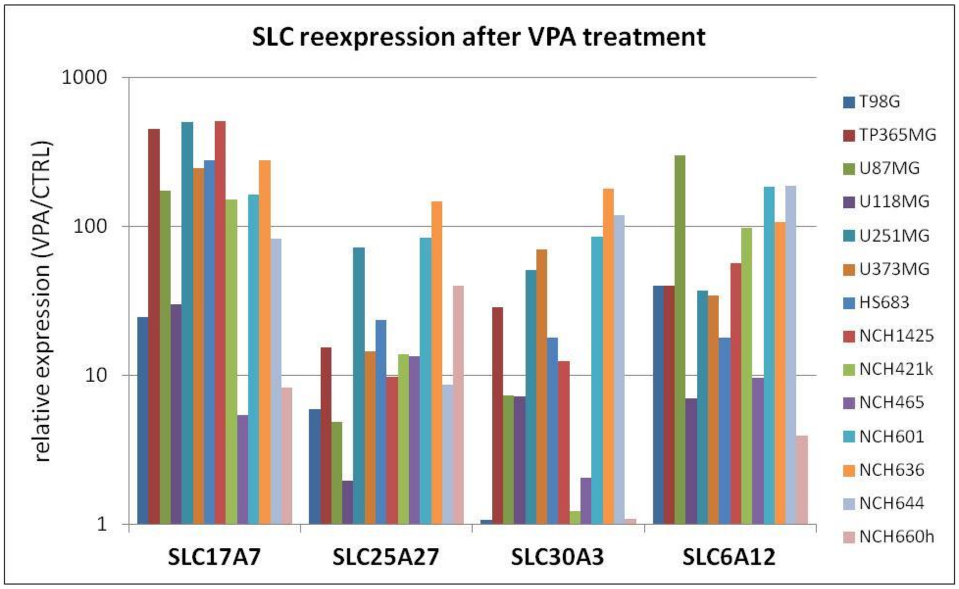

Figure S1: **Confirmation of SLC transporter reexpression after VPA treatment by real-time RT-PCR**. We exemplarily validated overexpression of the four transporters (SLC17A7, SLC25A27, SLC30A3 and SLC6A12) that had displayed the highest transcriptional upregulation on RNA-Seq.

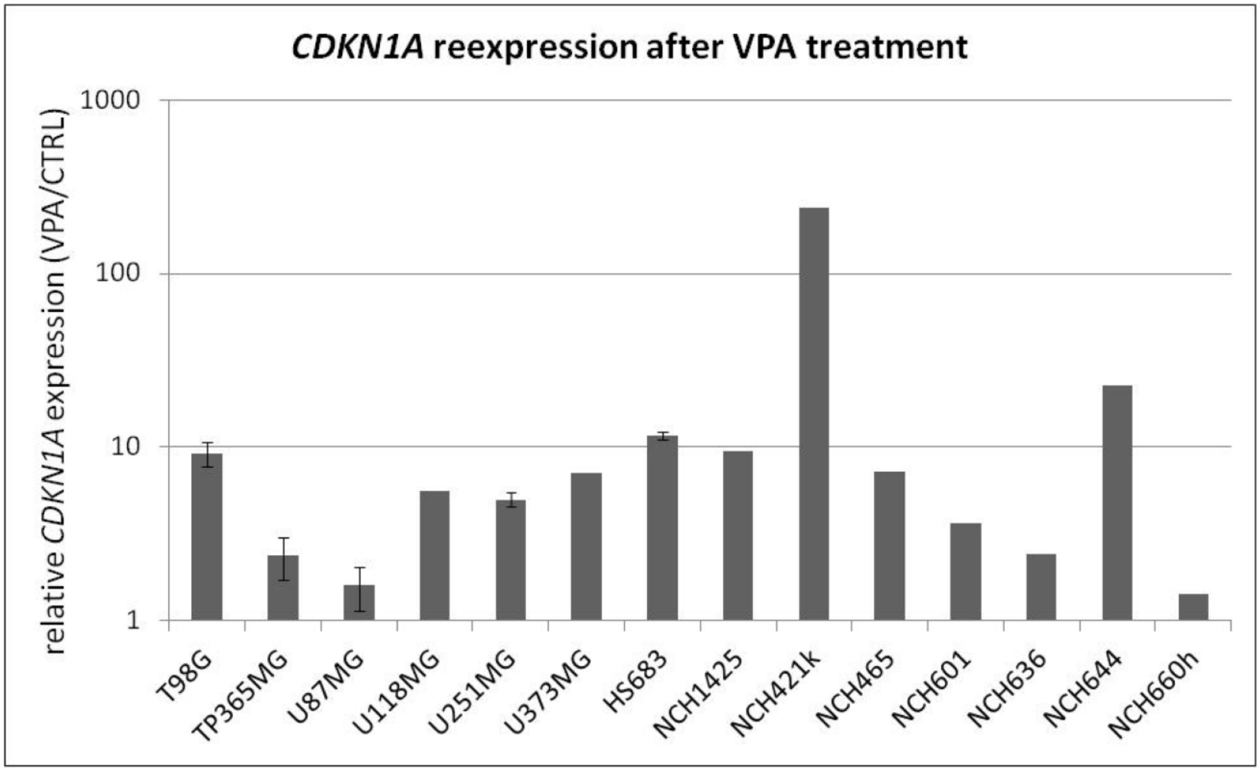

Figure S2: **Confirmation of reexpression of *CDKN1A* after VPA treatment by real-time RT-PCR.** These analyses confirm the upregulation observed on RNA-Seq with even all 14 cell lines displaying a VPA-induced transcriptional upregulation of cell cycle regulator *CDKN1A/p21* by this method.

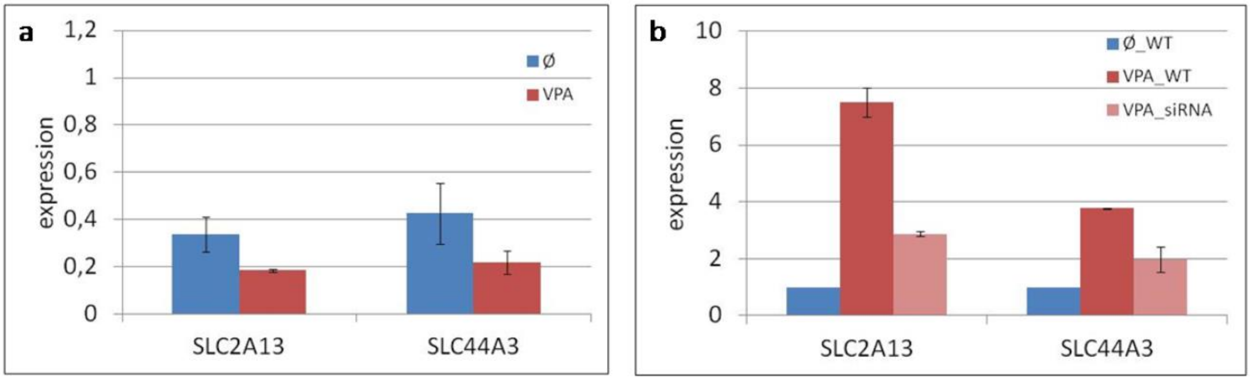

Figure S3: **siRNA-mediated silencing of SLC transporter expression**. (**a**) siRNA transfection itself worked sufficiently in the absence (blue) as well as in the presence (red) of VPA resulting in a reasonable knockdown in both situations (exemplarily analyzed for the SLC transporters SLC2A13 and SLC44A3). (**b**) VPA treatment (dark red) markedly increased SLC transporter expression compared to VPA-naive cells (blue). siRNA-mediated SLC transporter knockdown in VPA-treated cells (light red), however, did not suffice to decrease SLC transporter expression to the starting level measured in VPA-naive cells and therefore did only partially rescue the expression increase induced by VPA.
